# Discrimination Power of Short Essay Questions Versus Multiple Choice Questions as an Assessment Tool in Clinical Biochemistry

**DOI:** 10.7759/cureus.35427

**Published:** 2023-02-24

**Authors:** Basmah Eldakhakhny, Ayman Z Elsamanoudy

**Affiliations:** 1 Clinical Biochemistry, King Abdulaziz University Faculty of Medicine, Jeddah, SAU; 2 Medical Biochemistry and Molecular Biology, Mansoura University, Faculty of Medicine, Mansoura, EGY

**Keywords:** difficulty index, discrimination factor, clinical biochemistry, multiple-choice questions, short essay

## Abstract

Assessment is fundamental to the educational process. Multiple choice questions (MCQs) and short essay questions (SEQs) are the most widely used assessment method in medical school. The current study evaluated the discriminating value of SEQs compared to MCQs as assessment tools in clinical biochemistry and correlated undergraduate students' SEQ scores with their overall scores during the academic years 2021-2022 and 2022-2023. This is a descriptive-analytical study in which MCQ and SEQ papers of clinical biochemistry were analyzed. The mean score for SEQs in males was 66.7 ± 1.2 and for females it was 64.0 ± 1.1 SEM, with a p-value of 0.09; for MCQs, the mean score for males was 68.5 ± 0.9 SEM and for females it was 72.6 ± 0.8. When analyzing the difficulty index (DI) and discrimination factor (DF) of the questions, MCQs have a mean DI of 0.70 ± 0.01,and DF of 0.05 to 0.6. SEQs have a mean DI of 0.73 ± 0.03 and DF of 0.68 ± 0.01; there was a significant difference between the DF of MCQs and SEQs (p < 0.0001). Furthermore, there was a significant difference between SEQs and MCQs when categorizing students based on their scores, except for A-scored students. According to the current study, SEQs have a higher discriminating ability than MCQs and help differentiate high-achieving students from low-achieving students.

## Introduction

Assessment is fundamental to the educational process. It has benefits beyond measuring knowledge and competence alone. It is also crucial for directing and stimulating the learning process, as well as providing feedback to teachers and learners [1]. The assessment of the competence of undergraduate medical students is a very crucial mission [2]. The following three learning domains are to be evaluated: knowledge and understanding, skill, and value. The skills' domain is assessed at the levels of comprehension, application, analysis, synthesis, and criticism [3].

There are many methods for assessing the skills domain. They include a free response examination, long essay questions, short essay questions (SEQs), modified essay questions, multiple choice questions (MCQs), and others. Each of these methods has its advantages and disadvantages. The evaluation method's reliability and validity necessitate combining these methods [2,3]. MCQs emphasize mainly on knowledge recall, level I of revised Bloom's taxonomy; they can also assess a higher cognitive level when properly constructed. SEQs are efficient in assessing higher-order thinking and are associated with item writing flaws [4,5].

Assessment can be formative and summative. Formative assessment helps teachers identify students' learning gaps and modify teaching strategies [6]. Summative assessment is done at midterm and final examinations in most medical schools [7]. In the Faculty of Medicine at King Abdulaziz University (KAU), Jeddah, Saudi Arabia, Clinical Biochemistry core course assessment tools are in the form of MCQs, SEQs, and practical examinations. The MCQs and SEQs are components of the written examinations (midterm and final examinations).

The study was conducted to compare and contrast the discriminating value of SEQs and MCQs as assessment tools in clinical biochemistry. Moreover, it aimed to correlate the students' scores of SEQs with their overall academic scores in the clinical biochemistry course at the Faculty of Medicine, KAU.

## Materials and methods

Setting

This study was conducted in the Clinical Biochemistry Department, School of Medicine, KAU, during the study years 2021-2022 and 2022-2023. It is a descriptive-analytical study in which MCQ and SEQ examinations of clinical biochemistry were analyzed. The ethical committee (Ethics Committee of Human Research at KAU) ruled that no formal ethics approval was required in this case.

Clinical biochemistry (BCHM 201) is a five-credit course for second-year medical students. The contact hours are in the form of four theoretical interactive lectures, one tutorial session, and one practical session per week. The students were familiarized with the assessment plan from day 1 of the course. The total score of the course (out of 100) was divided between different methods of assessments: 75% for written examinations (22.5% SEQs and 52.5% MCQs) which were in the form of midterm and final examinations, 10% for practical laboratory reports, 10% for final practical experiments, and the last 5% for team-based learning, with 60% as a passing grade. Students were divided into five groups based on their total scores in percentage: A for students who scored ≥ 90%, B for those who scored 80-89.99%, C for those who scored 70-79.99%, D for those who scored 60-69.99%, and F for those who scored < 60%.

Study protocol

In the current study, a total of 726 students' grades were analyzed, of whom 358 were male and 368 were female. The detailed scores were analyzed to study how students' achievement may differ between SEQs and MCQs. Each student's MCQ and SEQ scores were recorded, normalized to a percentage (%), and compared to the total score they received upon course completion. After each examination, an item analysis report with difficulty index (DI) and discrimination factor (DF) was created. For MCQs, item analysis was done using QuestionMark perception 5.7 software for Windows, whereas SEQ item analysis was done using the ZipGrade App Version 2.56. SEQs were marked, and the score was recorded on a ZipGrade answer sheet designed to record scores ranging from 0 to 3, with three counts as the primary answer and equal to 100% and zero counts as the lowest mark and equal to 0%. Any number in between is considered a partial answer, and students receive a percentage of the mark based on their score; the SEQs’ DF was calculated by ZipGrade using the Pearson correlation. Examinations were constructed based on the course learning outcome (CLO)-dependent blueprint and included MCQs and SEQs for each CLO from the knowledge and understanding as well as skills domain. All questions were reviewed by the examination committee members, including three professors and two associated professors of clinical biochemistry, to evaluate question quality, scientific information, and the English language. SEQs, as mentioned above, cover all CLOs and are constructed as figure interpretation, pathway completion, comparison, or written a directed brief description. Two raters (one male and one female faculty member) evaluated the SEQs as per the pre-formed standards and model answers.

Statistical analysis

Data analysis was conducted using SPSS Version 23 (IBM Corp., Armonk, NY) and GraphPad Prism Version 9.5 (GraphPad Software, San Diego, California). P-value was calculated using an individual t-test to compare the mean score for males and females, a paired t-test was used for individual students' scores (SEQs, MCQs, and total), and the Mann-Whitney U test was used to compare the MCQs and SEQs’ difficulty and discrimination. Pearson correlation coefficient (r) was computed to assess the linear relationship between the SEQs and MCQs in different groups. Data are presented as mean ± standard error of the mean (SEM), and a p-value was considered significant if it was <0.05.

## Results

**Figure 1:**
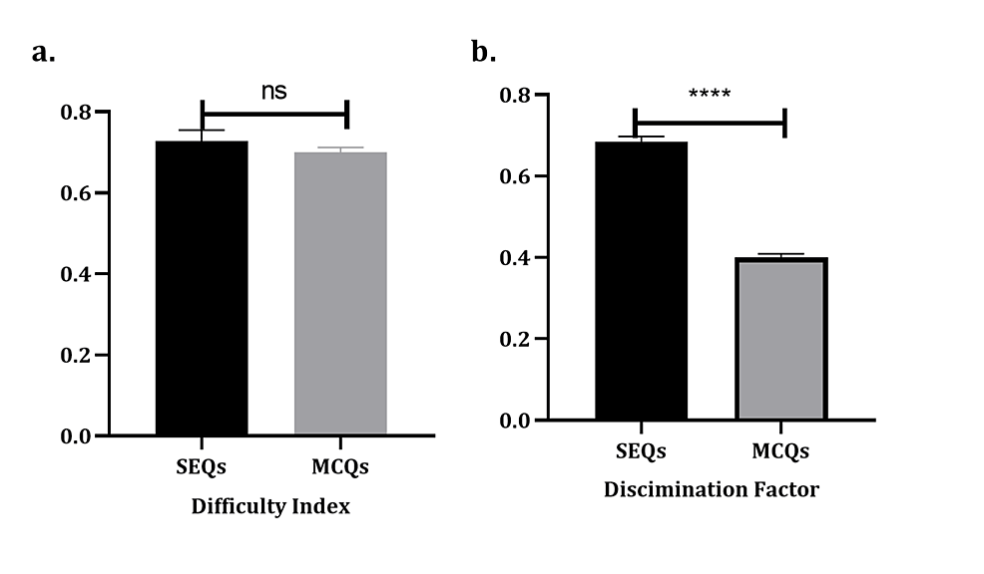
Comparison between MCQs and SEQs’ difficulty and discrimination. A total of 160 MCQs and 34 SEQs were analyzed. (a) There is no statistically significant difference in the difficulty of SEQs and MCQs. (b) The difference between the DF of SEQs and MCQs shows that SEQs have much higher discrimination with p < 0.0001 (as indicated by asterisks). The data are presented as mean ± SEM SEQ, short essay question; MCQ, multiple choice question; SEM, standard error of mean

The item analysis of 160 MCQs and 34 SEQs was conducted and is summarized in Table 1 and Figure 1. MCQs have a mean DI of 0.70 ± 0.01 SEM, with a minimum score of 0.25 and a maximum score of 0.98. Their DF ranged from 0.05 to 0.6, with an average of 0.40 ± 0.01. On the other hand, the DI of SEQs ranged from 0.4 to 0.95, with an average of 0.73 ± 0.03. Their DF ranged from 0.45 to 0.85, with an average of 0.68 ± 0.01. There was no statistical difference between the DI of MCQs and SEQs. In contrast, there was a statistical significance with a p-value of <0.0001 for the DF.

**Table 1:** Comparison between MCQs and SEQs’ difficulty and discrimination. P-value was calculated using the Mann-Whitney U test. Data are presented as mean ± SEM. SEQ, short essay question; MCQ, multiple choice question; SEM, standard error of mean

	SEQs, n = 34 (mean ± SEM)	MCQ, n = 160 (mean ± SEM)	P-value
Difficulty index	0.73 ± 0.03	0.70 ± 0.01	0.3639
Discrimination factor	0.68 ± 0.01	0.40 ± 0.01	<0.0001

**Figure 2:**
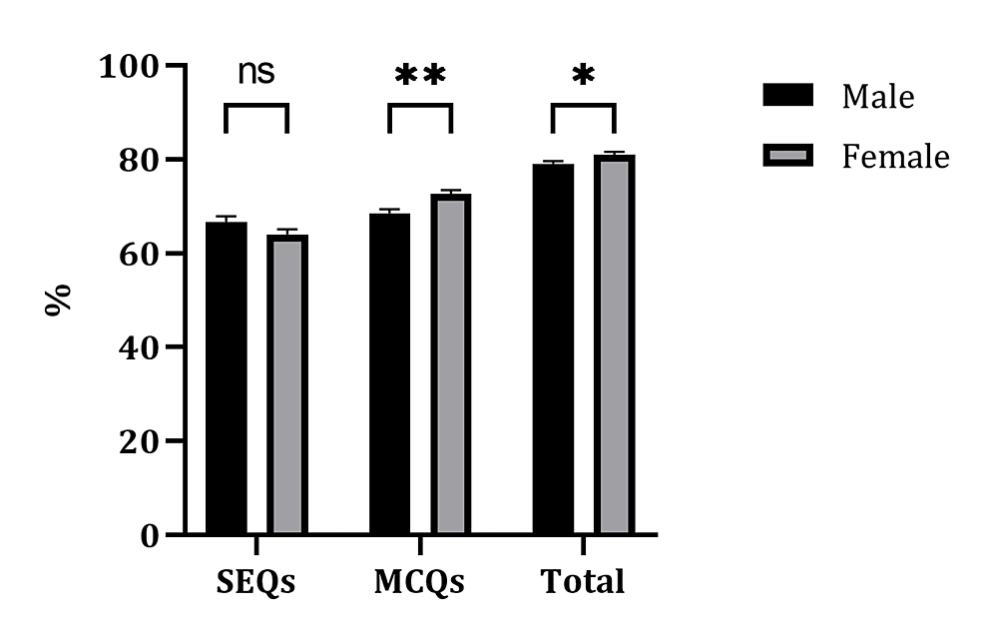
A comparison of SEQs, MCQs, and totals scores between males and females. A total of 726 students' grades were analyzed, of whom 358 were male and 368 were female. Data showed statistical significance in MCQs and total scores. Data are presented as mean ± SEM. **P-value of 0.001. *P-value of 0.02. SEQ, short essay question; MCQ, multiple choice question; SEM, standard error of mean

A total of 726 students' grades were analyzed, of whom 358 were male and 368 were female. The mean score for SEQs in males was 66.7 ±1.2 SEM and for females it was 64.0 ± 1.1 SEM, with a p-value of 0.09. For MCQs, the mean score for males was 68.5 ± 0.9 SEM and for females it was 72.6 ± 0.8 SEM, with p < 0.001. Finally, the total mean scores were 79 ± 1 SEM in males and 81 ± 1 in females, with a p-value of 0.02. Data can be seen in Figure 2.

**Figure 3:**
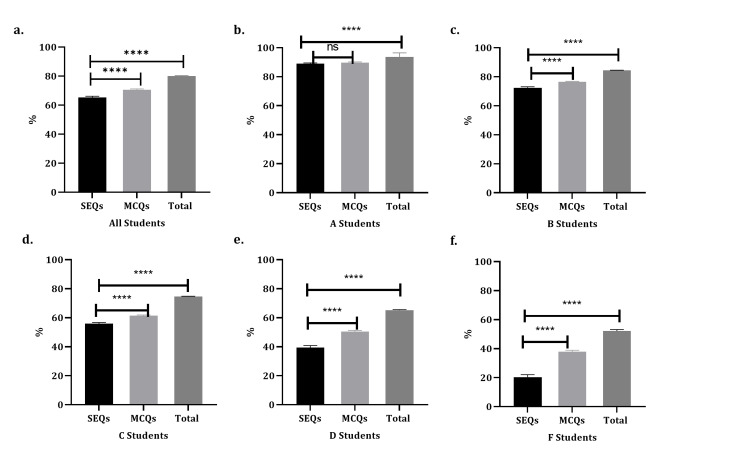
Comparison of SEQs, MCQs, and total scores in different grade groups. The overall scores of 726 students were divided into five groups (A-F), and the means of their SEQs, MCQs, and total scores were compared. A-F show that the difference in all students and each group between SEQs, MCQs, and totals scores are all statistically significant except for group A. Data are presented as mean ± SEM. ****p-value < 0.0001. SEQ, short essay question; MCQ, multiple choice question; SEM, standard error of mean

Students were further classified into A-F groups based on their overall course grades, and their SEQ scores were compared to their MCQ and total scores. Group B had the highest number of students (258), while group F had the lowest (47). Interestingly, looking at the average grade of all students, there was a 5% difference between the mean SEQ and MCQ scores, and almost a 15% difference between SEQs and total scores, with the higher grade in MCQs and total scores compared to SEQs. These differences are not statistically significant in students with average grades A. However, the gap keeps increasing in students with lower scores (group F). The difference reaches 15 % between SEQs and MCQs and 30% between SEQs and the total scores. These comparisons are detailed in Table 2 and Figure 3. Finally, a Pearson correlation coefficient (r) was calculated, as shown in Table 3, to determine whether there was a linear relationship between SEQs, MCQs, and total grades. When looking at overall students, there was a strong positive correlation between SEQ scores and both MCQ (r = 0.85 and p < 0.0001) and total grades (r = 0.91 and p < 0.0001). Interestingly, this correlation becomes weak when looking at the group individually, ranging from 0.27 to 0.38, between SEQs and MCQs, although it is statistically significant, and ranging from 0.58 to 0.65 between SEQs and total score. Only group D had an r of 0.18, with no significance.

**Table 2:** Comparison of SEQs, MCQs, and total scores in different grade groups. P-value was calculated using paired t-test. SEQs. Data are presented as mean ± SEM. SEQ, short essay question; MCQ, multiple choice question; SEM, standard error of mean

	Count (n)	SEQs (mean ± SEM)	MCQs (mean ± SEM)	P-value (SEQs vs MCQ)	Total (mean ± SEM)	P-value (SEQs vs total)
All students	726	65.3 ± 0.8	70.6 ± 0.6	<0.0001	80.01 ± 0.44	<0.0001
A	169	88.95 ± 0.63	89.77 ± 0.44	0.2187	93.57 ± 0.22	<0.0001
B	258	72.47 ± 0.65	76.43 ± 0.45	<0.0001	84.38 ± 0.19	<0.0001
C	172	55.84 ± 0.85	61.36 ± 0.58	<0.0001	74.59 ± 0.25	<0.0001
D	80	39.36 ± 1.43	50.36 ± 0.9	<0.0001	65.3 ± 0.3	<0.0001
F	47	20.36 ± 1.72	37.77 ± 1.07	<0.0001	52.13 ± 1.05	<0.0001

**Table 3:** Correlation between SEQs, MCQs, and total scores. r: Pearson correlation coefficient SEQ, short essay question; MCQ, multiple choice question

SEQs %	Count (n)	MCQ %		Total course grade %	
		r	P-value	r	P-value
All students	726	0.852663	<0.0001	0.9131	<0.0001
A	169	0.27447	<0.0001	0.5867	<0.0001
B	258	0.303399	<0.0001	0.5497	<0.0001
C	172	0.306831	<0.0001	0.5245	<0.0001
D	80	0.188549	0.09	0.3771	0.0006
F	47	0.381957	0.008	0.6572	<0.0001

## Discussion

The current study was designed to assess the use of SEQs (structured essay type and short answer questions) in addition to MCQs as an assessment method for higher cognitive skills in clinical biochemistry.

MCQs have high reliability when the set of questions is valid with sufficient numbers of questions applied [8,9]. From our point of view, MCQs alone are not enough. They must be combined with another type of assessment to test the higher cognitive function.

MCQs are applicable for evaluating knowledge, understanding, and conception of factual information (the lower levels of cognitive processing) [10]. It can be constructed to measure application and analysis, but it requires a level of experience in formulating questions to measure a higher level of cognition [11]. MCQs are widely used due to their high reliability, validity, and ease of counting [12]. On the other hand, SEQs are structured in an open-ended format and intended to increase reproducibility and objectivity [9]. They are more complex, requiring students to recall facts and use higher-order cognitive skills [13]. It encourages the students to develop and use their critical thinking capabilities [14] besides their ability to enable the student to think and come to a conclusion about the answer, which is not a benefit in MCQs [15]. Therefore, it provides more benefits for assessment despite the time consumption during its preparation and scoring [4].

In the current study, the SEQs are more discriminating than MCQs, even though there is no statistically significant difference between the two types of questions in their DI scores.

Kunjappagounder et al. [16] reported that a well-framed essay question is an efficient tool for evaluating students' levels in the cognitive domain [16]. It is more discriminating than multiple questions. Our study's results are in agreement with those in the studies by Jaleel et al. (2020) [17], Kunjappagounder et al. [16], and Maryani et al. [18].

Evaluating questions item analysis is very important for assessing the SEQs. It consists of the analytical study of the individual questions and the whole test [19]. Measuring the degree of difficulty and discrimination is necessary for the reliability and validity of the assessment [16].

Item analysis is used for evaluating the degree of understanding by the students as well as providing feedback to the examiner. The most important indices are discrimination and difficulty indices. The DF shows the ability of a question to differentiate between a higher- and a lower-ability student [19-21].

The mean score of MCQs is much higher than that of the SEQs in the present study, which is consistent with the results of many earlier studies by Oyebola et al. [22], Wilkinson and Shaw [23], and Aalaei et al. [24].

Aalaei et al. [24] reported a similar finding; good discrimination is documented in the essay question more than in the multiple choice type [24]. Many factors can explain this finding, including guessing and the presence of the correct answer between the distractors, which can make them easier to be answered. However, the level of thought and concentration applied to the essay questions has a significant impact on distinguishing high achievers from lower achievers [24].

Moreover, SEQs necessitate students to interpret and analyze their thoughts to provide the answers; they also evaluate written communication skills [17].

This finding could show that any student with a reasonable degree of conceptual knowledge of the subject can score well in MCQs regardless of the depth of their understanding, which does not apply to essay-type questions. Consequently, the teaching process and examination model must highlight the deep learning approaches and structure concepts rather than memorization learning.

The high discriminating value of the essay question is also confirmed by the finding of the current study when categorizing the grades into A to F, which showed that the low achievers (F grades students) have a 30% gap between their MCQs and total scores in comparison to their SEQ scores.

As expected, the present study revealed positive linear correlations between SEQs, MCQs, and total grades. These correlations were also reported in other subjects, such as pharmacology [25], otorhinolaryngology [26], pediatrics [27], and basic medical sciences, including physiology and medical biochemistry [4,17].

However, the observed weak correlations in the low achiever grades (F) with non-significant correlation in D scorers confirm the previous concept of the higher discrimination ability of the essay question over the MCQs.

The limitation of the current study is the low number of SEQs compared to the MCQs. This is due to examination time limitations, as answering SEQs requires a longer duration than that for MCQs. Therefore, in this study, the proportion of the SEQs is much low in relation to the whole examination.

## Conclusions

We conclude that when comparing MCQs and SEQs (the structured and the open-ended ones) with the same level of cognition, SEQs have a higher discriminating ability and are helpful in differentiating high scorers from low scorers. Moreover, it is a good tool for assessing the high cognitive function regarding analysis and building concepts. Therefore, it is recommended to increase the percentage of the SEQs in the summative examinations and apply other types, such as extended essays, modified essays, and constructed-response questions. Moreover, we recommend using these questions in different branches of basic medical science.
